# Evolution of major histocompatibility complex class I and class II genes in the brown bear

**DOI:** 10.1186/1471-2148-12-197

**Published:** 2012-10-02

**Authors:** Katarzyna Kuduk, Wiesław Babik, Katarzyna Bojarska, Ewa B Śliwińska, Jonas Kindberg, Pierre Taberlet, Jon E Swenson, Jacek Radwan

**Affiliations:** 1 Institute of Environmental Sciences, Jagiellonian University, Gronostajowa 7, Kraków, 30-387, Poland; 2 Institute of Systematics and Evolution of Animals, Polish Academy of Sciences, Sławkowska 17, Kraków, 31-016, Poland; 3 Institute of Nature Conservation, Polish Academy of Sciences, Mickiewicza 33, Kraków, 31-120, Poland; 4 Department of Wildlife, Fish, and Environmental Studies, Swedish University of Agricultural Sciences, Umeå, SE, 901 83, Sweden; 5 Laboratoire d’Ecologie Alpine (LECA), Génomique des Populations et Biodiversité, CNRS UMR 5553, Université Joseph Fourier, BP 53, Grenoble Cedex 9, F-38041, France; 6 Department of Ecology and Natural Resources Management, Norwegian University of Life Sciences, Ãs, NO-1432, Norway; 7 Norwegian Institute for Nature Research, Trondheim, NO-7485, Norway

**Keywords:** Positive selection, Antigen binding sites, MHC gene expression, Phylogenetic analysis, Orthology, *Ursidae*

## Abstract

**Background:**

Major histocompatibility complex (MHC) proteins constitute an essential component of the vertebrate immune response, and are coded by the most polymorphic of the vertebrate genes. Here, we investigated sequence variation and evolution of MHC class I and class II DRB, DQA and DQB genes in the brown bear *Ursus arctos* to characterise the level of polymorphism, estimate the strength of positive selection acting on them, and assess the extent of gene orthology and trans-species polymorphism in *Ursidae*.

**Results:**

We found 37 MHC class I, 16 MHC class II DRB, four DQB and two DQA alleles. We confirmed the expression of several loci: three MHC class I, two DRB, two DQB and one DQA. MHC class I also contained two clusters of non-expressed sequences. MHC class I and DRB allele frequencies differed between northern and southern populations of the Scandinavian brown bear. The rate of nonsynonymous substitutions (d_N_) exceeded the rate of synonymous substitutions (d_S_) at putative antigen binding sites of DRB and DQB loci and, marginally significantly, at MHC class I loci. Models of codon evolution supported positive selection at DRB and MHC class I loci. Both MHC class I and MHC class II sequences showed orthology to gene clusters found in the giant panda *Ailuropoda melanoleuca*.

**Conclusions:**

Historical positive selection has acted on MHC class I, class II DRB and DQB, but not on the DQA locus. The signal of historical positive selection on the DRB locus was particularly strong, which may be a general feature of caniforms. The presence of MHC class I pseudogenes may indicate faster gene turnover in this class through the birth-and-death process. South–north population structure at MHC loci probably reflects origin of the populations from separate glacial refugia.

## Background

The major histocompatibility complex (MHC) is a key element of the vertebrate immune system, responsible for presentation of foreign peptides to T-cells [1]. MHC consists of two main groups of genes, MHC class I and MHC class II, each comprising a number of genes that appear to evolve by the birth-and-death process, whereby some new genes appear via duplication and others are pseudogenised or deleted [2,3]. MHC class I genes are expressed in all nucleated cells and present antigens derived mostly from intracellular parasites, whereas MHC class II genes are expressed in specialised antigen presenting cells, such as macrophages, and present mostly antigens of extracellular parasites. The peptide-binding groove of class I molecules is formed by α_1_ and α_2_ chains encoded by the second and third exon of the gene, whereas class II peptide binding groove is formed by α and β chains encoded by second exons of separate A and B genes [4].

MHC genes are the most polymorphic genes described in vertebrates, with polymorphism occurring predominantly at residues involved in peptide binding (antigen binding sites, ABS; [5-7]). The mechanisms deemed responsible for maintaining polymorphism at MHC genes include frequency-dependent selection [8,9] and heterozygote advantage [10]. Frequency dependence arises because the bearers of common alleles become more likely to be evaded by evolving parasites (e.g. [11]), whereas heterozygosity allows presentation of a wider range of pathogen-derived peptides, and thus provides better resistance to infection (e.g. [12]). Consistent with evolution under pressure from parasites, there is a growing evidence for an association between MHC types and susceptibility to parasites (e.g. [13-22]). Additionally, several taxa have been shown to avoid mating with MHC-similar partners (e.g. [23-26]), and such MHC-disassortative mating should also help maintain MHC polymorphism [27]. Balancing selection acting on MHC appears to be able to maintain allelic lineages for a long time, resulting in trans-species polymorphism, whereby some alleles from different species are more similar than some alleles within species [28].

The mechanisms of balancing selection summarised above not only maintain polymorphism, but also favour novel alleles with non-synonymous substitutions changing peptide-binding properties of MHC molecules. Indeed, the rates of non-synonymous substitutions often exceed those of synonymous substitutions at sites involved in peptide binding [29-32].

The rate of birth-and-death process appears faster in MHC class I genes than in class II loci, and as a result it is difficult to establish orthology of class I genes among mammalian orders [31,33]. In contrast, clusters of MHC class II genes, which originated 170–200 mya, retain orthology between orders of mammals [34]. The reasons for this difference between class I and class II genes are not well understood. In humans, differences in the rate of the birth-and-death process is not mirrored in the differences in the strength of positive selection, as d_N_/d_S_ ratios are very similar for both MHC classes, but in mice d_N_/d_S_ in MHC class I genes is considerably lower than that in MHC class II [29,31].

Here, we characterised sequences of 2^nd^ exon of the MHC class I and class II genes in the Scandinavian population of brown bear *Ursus arctos*. The 2^nd^ exon encodes one of two chains forming the peptide binding groove in both MHC classess. MHC class II genes in the brown bear from Japan have been studied by Goda *et al*. [35,36], who found considerable polymoprhism of DRB genes, but limited polymorphism of DQA genes. MHC class I in the brown bear has not been characterised so far. Our study had the following aims: (i) to characterise the level of polymorphism of both MHC classes, (ii) to compare the strength of positive selection acting on them, based on the patterns of nucleotide substitution, and (iii) to assess the extent of gene orthology and trans-species polymorphism between the brown bear and the giant panda *Ailuropoda melanoleuca*. Based on generally higher rate of evolution of class I genes among mammals, we expected the extent of trans-species polymorphism to be lower for class I genes. Due to excellent long-term data about mating patterns, reproductive success and parasite load, the Scandinavian brown bear is an ideal system to study contemporary selection on MHC resulting from parasites and mate choice. The present study provides a basis for such work.

## Methods

### Samples

Samples analyzed in the present study originated from two brown bear populations sampled within the Scandinavian Brown Bear Research Project. The northern population (N) is located near Jokkmokk in Norrbotten County, Northern Sweden, and the southern population (S) consisted of samples collected in Dalarna and Gävleborg counties in Central Sweden and Hedmark County in Southeastern Norway [37]. Details on sampling and genomic DNA (gDNA) extraction can be found in Waits *et al*. [38] and Bellemain *et al*. [39]. Altogether samples from 234 individuals were used to characterize polymorphism in MHC and 6 samples from the southern population were used to characterize expression of four MHC genes. Samples were collected under permissions: C7/12 for 2012–2014 and C47/9 for 2009–2012, C59/6 for 2006–2008, C40/3 for 2003–2006 from Uppsala Ethical Committee on Animal Experiments, Uppsala, Sweden.

### Primer development

Comprehensive characterization of variation in highly polymorphic MHC genes requires PCR primers amplifying all alleles. To develop such primers for the second, most variable, exon of several MHC genes, we used two approaches. The first was based on the vectorette PCR and the second employed primers located in conserved portions of exons 1 and 3 or 4 to amplify the intervening fragments from cDNA.

### Vectorette PCR

We designed primers in conserved regions of the second exon, identified in the alignment of mammalian MHC sequences downloaded from GenBank (Additional file 1:Table S1, the list of accession numbers in Additional file 2: Supplementary information). Parts of the second exon for all four genes were amplified from several individuals, cloned and sequenced as described in Zagalska-Neubauer *et al*. [40]. These partial sequences allowed the design of several primers within the second exon, which were used in vectorette PCR, performed as described in Babik *et al*. [41], to obtain sequences of 5’ and 3’ ends of the second exon from multiple individuals. In the vectorette PCR approach, total genomic DNA is digested with a restriction enzyme (RE) producing sticky ends; then double-stranded adapters (vectorettes) matching the overhangs but showing some internal mismatch (‘bubble’) are ligated. By using one primer specific to the sequence in question and the other specific to the reverse complement of one of the vectorette strands (in the region of mismatch), it is possible to directionally amplify the genomic fragment between the specific primer and the RE recognition site, i.e. outside of the region of known sequence. The consensus of these sequences and sequences obtained from cDNA (see below) allowed the design of robust primers for all studied genes.

### cDNA analysis

We designed primers in conserved regions of the first and fourth (MHC class I genes, Table 1 – primer pair no. 1) or third (MHC class II genes, Table 1 – primer pairs no.: 2–4) exon using mammalian sequences downloaded from GenBank (Table 1, the list of accession numbers in Additional file 2: Supplementary information). Total RNA was extracted with PAXgene Blood RNA kit (Qiagen) from six blood samples frozen in Qiagen’s PAXgene Blood RNA tubes following the manufacturer’s protocol. Complementary DNA (cDNA) was obtained using Omniscript reverse transcriptase (Qiagen) and Oligo(dT)12-18 primer (Invitrogen). Fragments of MHC genes were amplified from cDNA in 15 μL mixes containing 7.5 μL of HotStarTaq Master Mix (Qiagen), 2 μM of each forward and reverse primers, 5.4 μL of PCR-grade water and 1.5 μL of cDNA template. The polymerase chain reaction (PCR) cycling scheme was as follows: 95°C for 15 min, 28 cycles of 95°C for 30 s, 57°C for 30 s, 72°C for 1 min, and the final elongation step at 72°C for 10 minutes. cDNA amplicons were pooled separately for each gene, cloned and 13 – 28 clones per pool were sequenced. Full second exon sequences were used in combination with sequences obtained with the vectorette PCR technique to design primers used in actual genotyping (Table 1 – primer pairs no.: 5–8). All newly designed primer pairs amplified the previously detected alleles, confirming the successful design of genotyping primers.

**Table 1:** Primer sequences for MHC in the brown bear

**Primer pair no.**	**Primer name**	**Primer sequence (5’-3’)**	**Gene**	**Amplicon size (bp)**
1	1UNIex1L	TCCTsCTGCTrCTsTCGG	MHC class I, 1^st^ exon	625
	1UNIex4R	GCCCAGCACCTCAGGGTG	MHC class I, 4^th^ exon	
2	2DRBUNIex1L	GAyryTGATGrTGCTGArC	MHC class II DRB, 1^st^ exon	343
	2DRBUNIex3R	CCAGGAGGkTGTGrTGCTGCA	MHC class II DRB, 3^rd^ exon	
3	2DQAUNIex1L	CCwsTGGArGTGAAGAsAT	MHC class II DQA, 1^st^ exon	255
	2DQAUNIex3R	AACACwGTsACCTCAGGAAC	MHC class II DQA, 3^rd^ exon	
4	2DQBUNIex1L	GGCTGAGGGCAGAGACTC	MHC class II DQB, 1^st^ exon	282
	2DQBUNIex3R	TGGAyGGGrAGATrGTCACTGT	MHC class II DQB, 3^rd^ exon	
5	URS_1_F	GCTCsCACTCCCTGAGGTAT	MHC class I,	228
	URS_1_R	CCwCGCTCTGGTTGTAGTA	2^nd^ exon	
6	URS_DRB_F_3	TTCACCAACGGsACGGAGCGG	MHC class II DRB,	192
	URS_DRB_R	CTTGTCGCyGCACCrkGAAGCT	2^nd^ exon	
7	URS_DQA_F	CTGACCATGTTGCTTACTACGG	MHC class II DQA,	202
	URS_DQA_R	CATTGGTAGCAGCGrTATAGTTGGA	2^nd^ exon	
8	URS_DQB_F	AGGATTTCGTGCTCCAGyTTAAG	MHC class II DQB,	224
	URS_DQB_R	CTCGCCGCTGCrGGATGArsCTG	2^nd^ exon	
9	URS_1_F	CGTATCGCCTCCCTCGCGCCATCAG	MHC class I, 2^nd^ exon	228
		xxxxxxGCTCSCACTCCCTGAGGTAT		
	URS_1_R	CTATGCGCCTTGCCAGCCCGCTCAG		
		xxxxxxCCwCGCTCTGGTTGTAGTA		
10	URS_DRB_F_3	CGTATCGCCTCCCTCGCGCCATCAG	MHC class II DRB, 2^nd^ exon	192
		xxxxxxTTCACCAACGGsACGGAGCGG		
	URS_DRB_R	CTATGCGCCTTGCCAGCCCGCTCAG		
		xxxxxxCTTGTCGCyGCACCrkGAAGCT		

xxxxxx indicates the location of a 6-bp tag in the fusion primer sequences.

### Genotyping

Investigated MHC genes exhibited varying levels of polymorphism and, consequently, several techniques were used for their genotyping. DQA and DQB genes were slightly or moderately polymorphic so to characterise most variation present in these genes it was sufficient to genotype a relatively small sample by Single Strand Conformational Polymorphism (SSCP) or cloning and sequencing. Genotyping of highly polymorphic class I and class II DRB genes was performed for large samples of individuals by 454 sequencing. For all genes, expression was assessed through genotyping cDNA and gDNA from six individuals.

### SSCP and sequencing

PCR conditions for both DQA and DQB were: 95°C for 15 min, 28 cycles of 95°C for 30 s, 55°C for 30 s, 72°C for 1 min, and the final elongation step at 72°C for 10 minutes. The SSCP analysis was performed using GMA gels (Elchrom Scientific). We added 4.5 μL of PCR product to 9.5 μL of premix containing formamide and 10 mM sodium hydroxide, denatured it for 5 min at 95°C and immediately cooled it on ice. Electrophoresis was conducted in 1 x TAE buffer at 8°C, 4 V/cm, for 18 hours. Gels were stained with SYBR Gold Nucleic Acid Gel Stain (Invitrogen). Allele sequences were obtained by sequencing the bands excised from gels. Additional screening for variation in these genes was performed by cloning amplicons pooled from 20 individuals and sequencing multiple clones.

### 454 sequencing

454 sequencing was used for genotyping of highly polymorphic MHC class I and MHC class II DRB genes because initial tests with the SSCP technique resulted in complex, uninterpretable patterns. PCR amplification was conducted using fusion primers, which contained the 454 Titanium adapter sequence (A in forward, B in reverse primer) at the 5’ end, followed by a 6-bp tag (barcode), which distinguished amplicons obtained in different PCR reactions, and the gene specific primer. Tag sequences differed from each other in at least three positions, which minimized the chance of misassigning sequencing reads due to errors in tag sequences. Sequences of fusion primers are given in Table 1 (primer pairs no. 9 and 10). Amplification was carried out in 15 μL, as described above. Ten individuals were amplified and sequenced twice to estimate the genotyping error. PCR products were pooled in approximately equimolar quantities, pools were purified with the MinElute PCR Purification Kit (Qiagen) and sequenced at the Functional Genomics Center Uni/ETH in Zurich. Sequencing was performed bidirectionally using the GS FLX Titanium MV emPCR kit for emulsion PCR and the GS FLX Titanium Sequencing Kit XLR70 in combination with GS FLX Titanium PicoTiterPlate Kit 70 x 75 for sequencing (Roche Applied Sciences). Extraction of reads from multifasta files, assignment of reads to individuals and generation of alignments of variants present in each amplicon were performed with jMHC software [42]. The output from jMHC was analysed using BLAST, Excel and Bioedit [43].

### Allele validation

To minimize the occurrence of false alleles that may be the artefacts of PCR or cloning, we followed the guidance of Lenz & Becker [44]. The artefacts that occur in 454 output may be divided into three types: i) substitutions caused by polymerase errors during PCR, ii) PCR-chimeras and iii) insertions, deletions and substitutions due to 454 sequencing errors [45-47]. The first two types are not specific to 454 sequencing and their frequency may be reduced at the PCR level [44]. Whereas the point substitutions and insertions – deletions (indels) should be relatively rare, the chimeras may be easily produced during PCR by recombination between true alleles [44,46]. Furthermore, some chimeras may have sequences identical to true alleles, because the latter may originate through historical recombination from other alleles. Distinguishing between the PCR chimeras and the true alleles is based on the rationale that chimeras should always occur with both parental alleles in the amplicon and that the artefacts should be less frequent, as measured by the number of reads per amplicon, than the true alleles.

True alleles were distinguished from the artefacts following the procedure described in Zagalska-Neubauer *et al.*[40] and Radwan *et al*. [48]. Briefly, for each sequence variant, we calculated the maximum per amplicon frequency (MPAF) in the whole dataset. Sequences were sorted according to their MPAF. Starting from arbitrary MPAF of 1.5% for MHC class I sequences and 3% for MHC class II DRB sequences, 65 and 28 sequence variants, respectively, were chosen to evaluate whether they represent true alleles or sequence artefacts (see Radwan *et al*. [48] for details). For MHC class I sequences within the 1.5-2% MPAF interval, 95% (19 of 20) variants were classified as artefacts and within 2-3%, 75% (8 of 12) were classified as artefacts. All of 33 variants with MPAF above 3% were classified as true alleles. MPAF for the least abundant true allele and most abundant artefact (1.53%-2.86%) defined the “grey zone”, which required a decision about whether a sequence was a true allele or an artefact on a case-by-case basis [48]. For MHC class II DRB sequences, all 12 variants in the 3-10% MPAF interval were classified as artefacts. All of 16 variants with MPAF above 10% were classified as true alleles.

Alleles were named according to the nomenclature proposed by Klein *et al*. [49] and were numbered in ascending order, starting from the most abundant one. We use the term “allele” for unique sequence variants for simplicity, but assigning sequence variants to loci was generally not possible in our study.

### Sequence analysis

Genetic differentiation between populations was measured by calculating pairwise F_ST_ in ARLEQUIN 3.5 [50]. Because assigning of alleles to loci was not possible, each allele was treated as a dominant locus, and binary encoded.

For all alleles, the average pairwise nucleotide distances (Kimura 2-parameter model - K2P), Poisson corrected amino acid distances, as well as the average rates of synonymous (d_S_) and nonsynonymous (d_N_) substitutions, using the Nei-Gojobori method [51] with the Jukes-Cantor correction for multiple substitutions, were computed in MEGA5 [52]. Standard errors were obtained through 1000 bootstrap replicates.

We used two approaches to test whether positive selection shaped the evolution of the second exon of the investigated genes; the one-tailed *Z* test comparing d_N_ to d_S_ and comparison of the likelihoods of codon-based models of sequence evolution. The Z-test, as implemented in MEGA5, compared the rates of synonymous vs. nonsynonymous substitutions at all codons, ABS and non-ABS. The location of putative ABS was inferred from the structure of human HLA genes [6]. For MHC class I, putative ABS location was conservatively inferred from the consensus of ABS common to human HLA-A, B and C genes. The comparison of models of sequence evolution for three genes (excluding MHC class II DQA) was performed in PAML 4.2 [53]. Three models were tested: i) M0: a single ω (d_N_/d_S_) for all codons, ii) M7: nearly neutral (0 < ω < =1), with ω variation approximated by β-distribution, iii) M8: positive selection (a proportion of codons with ω > 1), with ω variation approximated by β-distribution. The best fitting model was chosen on the basis of the lowest value of the Akaike Information Criterion (AIC, [54]). Positively selected codons under the M8 model were identified through the Bayes empirical Bayes procedure [55].

Phylogenetic trees visualizing similarities among MHC alleles were constructed under the Bayesian approach with mrBayes 3.1.2 software [56]. The general time-reversible (GTR) model of sequence evolution with the rate-variation (Γ) among sites was used; parameter values were estimated from the data. Priors were set to default values. Two independent Metropolis coupled Markov chain Monte Carlo simulations (four chains each, three of them heated, temp. 0.20) were run for five million generations and sampled every 1000 generations for DQA, DQB and DRB; for the class I longer runs of 20 million generations were necessary to reach convergence, trees were sampled every 2000 generations. The first 1000 (DQA, DQB, DRB) or 2000 (class I) trees were discarded as burn-in. To calculate the posterior probability of each bipartition, the majority-rule consensus tree was computed from the 8000 (DQA, DQB, DRB) or 16000 (class I) sampled trees.

## Results

### MHC class I

**Figure 1:**
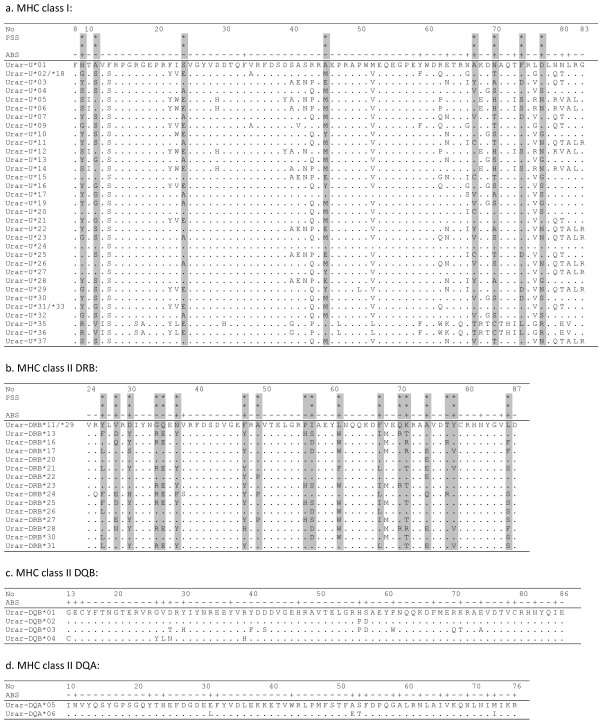
**Amino acid sequences of MHC alleles in the Scandinavian brown bear.** Positions in the alignment correspond to Human Leukocyte Antigen (HLA, after Reche & Reinherz, 2003). Dots mark identity to the reference sequence. The putative Antigen Binding Sites consensus positions (HLA-A, HLA-B and HLA-C for MHC class I 2^nd^ exon alleles, HLA-DRβ for MHC class II DRB 2^nd^ exon, HLA-DQα for MHC class II DQA 2^nd^ exon and HLA-DQβ for MHC class II DQB 2^nd^ exon; after Reche & Reinherz, 2003) are marked with ‘+’. Positively selected amino acid sites identified by the Bayes empirical Bayes procedure are shaded (** - posterior probability > 99%, * - posterior probability >95%).

We genotyped 228 bp fragment of MHC class I 2^nd^ exon in 224 bears, 100 from the northern and 124 from the southern population, and identified 37 alleles (GenBank accession numbers JX469853-89). The nucleotide sequences translated into 33 unique amino-acid sequences (Figure 1a). Two alleles had premature stop codons (PSC). Because assignment of alleles to loci was impossible, instead of true allele frequencies, frequencies of individuals possessing particular alleles are given in Table 2. Frequencies of MHC class I alleles differed significantly between the two Scandinavian brown bear populations (F_ST_ = 0.216, P < 10^-5^)

**Table 2:** Percentages of individuals possessing the particular MHC alleles in two populations of the Scandinavian brown bear

**Allele**	**North**	**South**
Urar-U*01	100	100
Urar-U*02	98	91
Urar-U*03	61	52
Urar-U*04	62	52
Urar-U*05	37	57
Urar-U*06	66	35
Urar-U*07	22	62
Urar-U*08	35	42
Urar-U*09	4	53
Urar-U*10	3	44
Urar-U*11	-	40
Urar-U*12	2	36
Urar-U*13	-	28
Urar-U*14	37	-
Urar-U*15	38	-
Urar-U*16	35	-
Urar-U*17	-	10
Urar-U*18	27	-
Urar-U*19	28	-
Urar-U*20	23	-
Urar-U*21	28	-
Urar-U*22	23	-
Urar-U*23	21	-
Urar-U*24	-	12
Urar-U*25	22	-
Urar-U*26	-	15
Urar-U*27	-	14
Urar-U*28	-	7
Urar-U*29	4	3
Urar-U*30	4	3
Urar-U*31	4	3
Urar-U*32	7	-
Urar-U*33	6	-
Urar-U*34	3	-
Urar-U*35	-	1
Urar-U*36	1	-
Urar-U*37	1	-
Urar-DRB*20	40	86
Urar-DRB*21	36	54
Urar-DRB*13	-	54
Urar-DRB*11	52	10
Urar-DRB*22	29	21
Urar-DRB*23	29	21
Urar-DRB*24	59	-
Urar-DRB*25	59	-
Urar-DRB*26	37	-
Urar-DRB*17	3	19
Urar-DRB*27	-	21
Urar-DRB*28	-	21
Urar-DRB*29	-	19
Urar-DRB*30	15	7
Urar-DRB*16	4	4
Urar-DRB*31	4	-

Alleles are sorted according to their total frequency.

**Figure 2:**
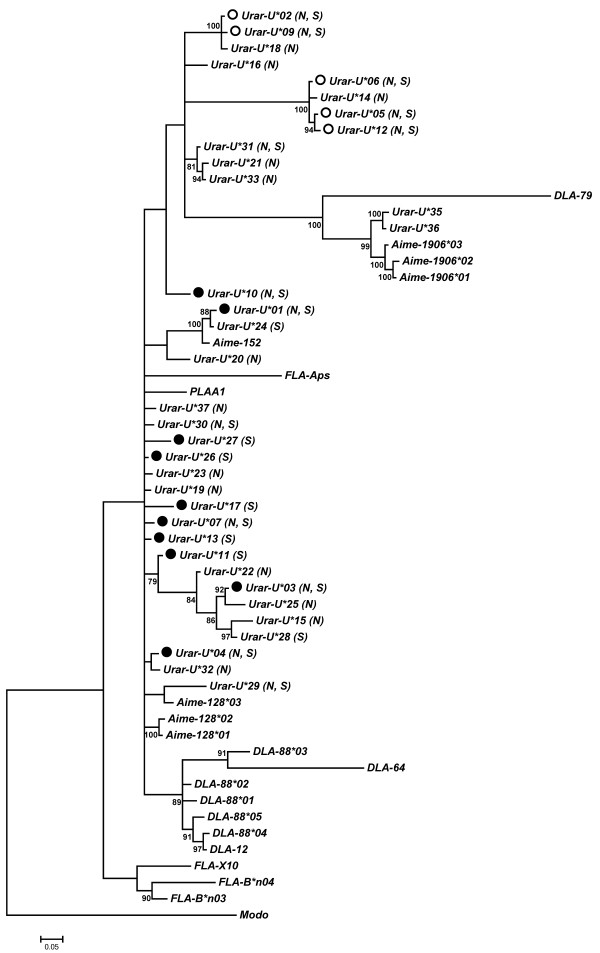
**Phylogenetic tree constructed for a 228 bp fragment of the second exon of MHC class I genes in the Scandinavian brown bear.** Bayesian posterior probabilities above 70% are shown above branches. Alleles marked with “S” – are present in the southern Scandinavian population, marked with “N” are present in the northern Scandinavian population. Alleles marked with filled circle are expressed; marked with open circle are putatively not expressed. The other sequences used to generate the tree are: *Ailuropoda melanoleuca*: *Aime-152* (EU162658.1), Aime-128*01 (EU162674.1), Aime-128*02 (EU162675.1), Aime-128*03 (EU162676.1), Aime-1906*01 (EU162662.1), Aime-1906*02 (EU162663.1), Aime-1906*03 (EU162664.1); *Canis lupus*: *DLA-12* (NM_001014379.1), *DLA-64* (NM_001014378.1), *DLA-79* (NM_001020810.1), DLA-88*01 (AF100567.1), DLA-88*02 (AF100568.1), DLA-88*03 (AF100569.1), DLA-88*04 (AF100570.1), DLA-88*05 (AF100571.1); *Felis catus*: *FLA-Aps* (M27192.1), FLA-B*n03 (EU915357.1), FLA-B*n04 (EU915358.1), *FLA-X10* (FCU0767388874.1); *Phoca vitulina*: *PLA-A1* (U88874.1). *Monodelphis domestica* sequence: *Modo* (NM_001044223.1) was used to root the tree.

A comparison of genotypes obtained from cDNA and gDNA of 6 individuals confirmed the expression of 10 alleles (Urar-U*01, *03, *04, *07, *10, *11, *13, *17, *26, *27), whereas 6 alleles were found only in gDNA (Urar-U*02, *05, *06, *08, *09, *12). The non-expressed alleles form two distinct clusters, consistent with the presence of two non-expressed MHC class I loci (Figure 2). We found 5–11 alleles per individual in gDNA. The allele Urar-U*01 was present in all individuals. The 6 bears assayed for MHC class I expression had 6–9 alleles in genomic DNA, but only 3–5 were detected in cDNA (Additional file 3: Table S2). This indicates the expression of at least 3 MHC class I loci in the brown bear, and the presence of a minimum of two non-expressed loci; at least one of these numbers is certainly an underestimate, because a minimum of six loci were present in gDNA when the entire population sample is considered. There was complete concordance between genotypes of both replicates in all cases.

Alleles Urar-U*35 and *36 group with *Aime-1906* locus of giant panda (Figure 2). Similar to the panda, alleles of this locus contain amino acid substitution at position 59, where Y is replaced by F, indicating non-classical nature of these alleles [57,58]. A similar substitution was found in Urar-U*02, *09 and *18 alleles, which form a separate, non-expressed cluster. Allele Urar-U*01 (present in all individuals) and the *24 group with *Aime-152* locus. This locus, monomorphic in giant panda, does not bear the landmarks of a non-classical gene [58].

Ninety of 228 (39.47%) nucleotide positions and 42 of 76 (55.26%) amino-acid positions were variable. Pairwise differences between alleles varied from 0.44% to 31.74% and amino-acid translations showed between 0 and 61.72% pairwise differences. Average nucleotide and amino-acid distances are listed in Table 3. Across all sites, d_N_ and d_S_ were similar and consequently d_N_/d_S_ did not differ significantly from 1 (Table 4). For ABS, however, d_N_ exceeded d_S_ by a factor of two, although the excess of non-synonymous substitutions was marginally non-significant. The model of codon evolution allowing for positive selection (M8) fitted the data better than models without positive selection (Table 5). The Bayes Empirical Bayes procedure identified eight codons under positive selection (positively selected sites, PSS; Figure 1), five of which were located at ABS, which is more than random expectation (Fisher’s exact p = 0.001).

**Table 3:** The average pairwise nucleotide and amino-acid distances for MHC class I and class II genes in the brown bear

	** K2P nucleotide distance**			** Poisson corrected amino acid distance**		
	**All sites**	**ABS**	**Non-ABS**	**All sites**	**ABS**	**Non-ABS**
MHC class I	14.7 (1.6)	44.8 (7.6)	10.6 (1.4)	25.0 (4.0)	87.0 (15.7)	17.5 (3.5)
MHC class II DRB	8.2 (1.4)	32.5 (6.5)	2.4 (0.8)	18.1 (3.9)	74.2 (15.6)	6.4 (2.3)
MHC class II DQA	3.0 (1.2)	12.0 (5.3)	0.0	6.2 (3.0)	25.1 (13.2)	0.0
MHC class II DQB	6.4 (1.3)	17.9 (4.6)	3.0 (1.0)	11.1 (3.0)	37.3 (11.7)	3.7 (1.9)

Distances and standard error values are given as percentages per site; standard errors, obtained through 1000 bootstrap replicates, are in parentheses. K2P, Kimura 2-parameter models.

**Table 4:** **The average rates of nonsynonymous (d**_**N**_**) and synonymous substitutions (d**_**S**_**) per**

		**d**_**N**_	**d**_**S**_	**Z**	**P**
MHC class I	All sites	15.1 (2.6)	13.7 (2.8)	0.439	0.331
	ABS	50.6 (11.5)	26.2 (11.1)	1.627	0.053
	non-ABS	10.1 (2.3)	12.0 (3.0)	−0.594	1.000
MHC class II DRB	All sites	10.2 (2.4)	2.6 (1.2)	**3.792**	**0.000**
	ABS	42.2 (9.4)	8.3 (4.4)	**4.320**	**0.000**
	non-ABS	2.9 (1.1)	1.1 (1.1)	1.536	0.064
MHC class II DQA	All sites	3.3 (1.7)	2.3 (2.4)	0.327	0.372
	ABS	12.5 (6.7)	10.2 (13.5)	0.144	0.433
	non-ABS	0.0	0.0	0.000	1.000
MHC class II DQB	All sites	6.5 (1.9)	6.3 (2.5)	0.071	0.472
	ABS	20.9 (6.8)	7.6 (5.8)	**1.967**	**0.026**
	non-ABS	2.0 (1.1)	6.1 (3.0)	−1.197	1.000

Distances and standard error values are given as percentages per site; standard errors obtained through 1000 bootstrap replicates in parentheses. Significant results are highlighted in bold.

**Table 5:** Evaluation of the fitting of models of codon evolution in the brown bear

**Model**	**ln L**	**Parameters**	**Δ AIC**
	MHC class I		
M0	−1725.4	ω = 0.474	313.2
M7	−1587.7		39.8
M8	−1565.9	p0 = 0.743 p1 = 0.257 ω_1_ = 2.325	0 – best
	MHC class II DRB		
M0	−694.5	ω = 2.327	117.1
M7	−646.7		23.6
M8	−632.9	p0 = 0.652 p1 = 0.348 ω_1_ = 12.181	0 – best
	MHC class II DQB		
M0	−402.8	ω = 0.322	9.5
M7	−397.1		0 – best
M8	−396.8	p0 = 0.717 p1 = 0.283 ω_1_ = 1.489	3.5

Parameter description : ω – d_N_/d_S_ per all sites; ω1 – estimated value of ω for sites under positive selection, p0 – proportion of sites with ω ≤ 1 ; p1 – proportion of sites with ω > 1.

Because in pseudogens the signal of positive selection may erode over time, we also carried out tests for positive selection after excluding sequences of pseudogens and non-classical MHC class I genes, but this did not change the results qualitatively (Additional file 4: Table S3).

### MHC class II DRB

We assayed a 192 bp fragment of the DRB second exon. Sixteen DRB alleles were identified among 234 individuals (100 from the north and 134 from the south) (Additional file 5: DRB sequences, these sequences did not reach the minimum of 200 bp required currently for GenBank submission). Four of these were identical to alleles reported by Goda *et al*. [36] (Urar-DRB*11, *13, *16, *17). The sequences did not contain indels or PSC. The 16 nucleotide sequences translated into 15 unique amino-acid sequences (Figure 1b). Frequencies of individuals possessing particular alleles are given in Table 2. F_ST_ between the north and south was 0.304 (P < 10^-5^).

**Figure 3:**
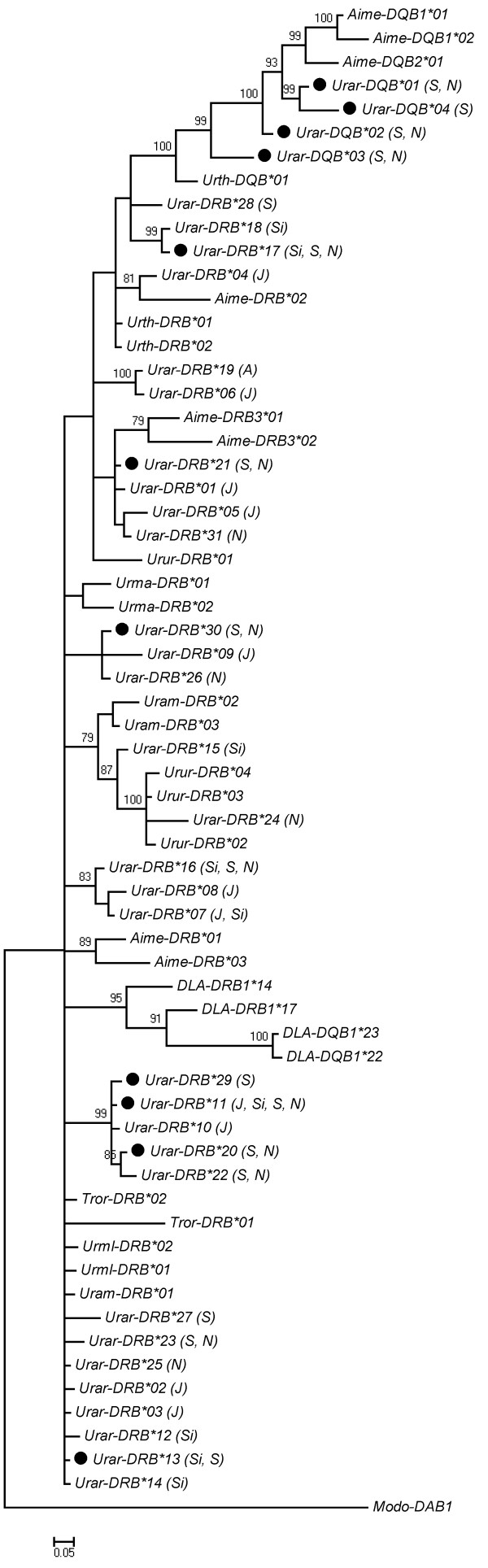
**Phylogenetic tree constructed for 189 bp long fragment of second exon of DRB and DQB genes in the brown bear.** Bayesian posterior probabilities above 70% are shown above branches. Alleles marked with “S” – are present in the southern Scandinavian population, marked with “N” are present in the northern Scandinavian population, ”J” – in Japan , “Si” - in Siberia, “A” – in Alaska [36]. Alleles marked with filled circle are expressed. The other sequences used to generate the tree are: *Ailuropoda melanoleuca*: Aime-DQB1*01 (GQ496182.1), Aime-DQB1*02 (GQ496183.1), Aime-DQB2*01 (GQ496188.1), Aime-DRB*01 (AY895155.1), Aime-DRB*02 (AY895156.1), Aime-DRB*03 (AY895157.1), Aime-DRB3*01 (GQ496165.1), Aime-DRB3*02 (GQ496166.1); *Ursus thibetanus japonicus*: Urth-DQB*01 (AB473936.1), Urth-DRB*01 (AB490478.1), Urth-DRB*02 (AB490479.1); *Ursus americanus*: Uram-DRB*01 (AB490480.1), Uram-DRB*02 (AB490481.1), Uram-DRB*03 (AB490482.1); *Ursus maritimus*: Urma-DRB*01 (AB490476.1), Urma-DRB*02 (AB490477.1); *Tremarctos ornatus*: Tror-DRB*01 (AB490489.1), Tror-DRB*02 (AB490490.1); *Melursus ursinus*: Urur-DRB*01 (AB490485.1), Urur-DRB*02 (AB490486.1), Urur-DRB*03 (AB490487.1), Urur-DRB*04 (AB490488.1); *Helarctos malayanus*: Urml-DRB*01 (AB490483.1), Urml-DRB*02 (AB490484.1); *Canis lupus*: DLA-DQB1*22 (AF113704.1), DLA-DQB1*23 (AF113705.1), DLA-DRB1*14 (U44779.1), DLA-DRB1*17 (U44780.1). *Monodelphis domestica* sequence: *Modo-DAB1* (NM_001032991.1) was used to root the tree.

Seven expressed alleles were found in six bears assayed for expression (Figure 3). Two to four alleles per individual were found in gDNA, all expressed, which implies the presence of at least two expressed loci. No discrepancies between replicates were found in 10 replicated individuals (maximum genotyping error 2.6%).

DRB alleles grouped with DRB of other ursids, and separately from DQB alleles (Figure 3). The phylogenetic tree did not reveal clusters corresponding to two loci inferred above: there were 3 well supported clusters, and relationships among the remaining alleles were poorly resolved (Figure 3).

Thirty-seven of 192 (19.27%) nucleotide and 21 of 64 (32.81%) amino-acid positions were variable. Pairwise nucleotide differences ranged from 0.52% to 16.87%, and amino-acid sequence differences ranged between 0 and 33.03%. Nucleotide and amino-acid distances are reported in Table 3. d_N_ significantly exceed d_S_ for all codons, and especially for ABS codons (by a factor of about 5); also at non-ABS sites d_N_ was higher than d_S_ (by a factor of nearly 3), but the difference was not significant (Table 4). PAML analysis showed the best fit of the positive selection model M8 (Table 5). The Bayes Empirical Bayes procedure identified 18 PSS (Figure 1), of which 12 were in ABS. The excess of PSS at ABS was significant (Fisher’s exact p < 0.0001).

### MHC class II DQB

Four alleles were found in the 224 bp fragment of the DQB 2^nd^ exon in 26 genotyped individuals (GenBank accession numbers JX469892-5), each translating into a unique amino-acid sequence (Figure 1c). The sequences did not contain indels or PSC. One to 3 alleles per individual were present and all of them appear expressed.

*Urar-DQB* 2^nd^ exon sequences formed a cluster separate from *Urar-DRB* sequences, but grouped with giant panda DQB (Figure 3). However, brown bear sequences were more similar to each other than to any of the giant panda DQB sequences.

Twenty-five of 224 (11.16%) nucleotide and 14 of 74 (18.92%) amino-acid positions were variable. Pairwise differences ranged between 2.27% and 11.04% for nucleotide sequences, and pairwise amino-acid differences ranged between 2.74% and 20.97%. Nucleotide and amino-acid distances are presented in Table 3. Across all sites, d_N_ was nearly equal to d_S_, but for ABS d_N_ was significantly higher (by a factor of about 3; Table 4). However, PAML analysis did not provide evidence for positive selection, as M7 model fitted the data best.

### MHC class II DQA

**Figure 4:**
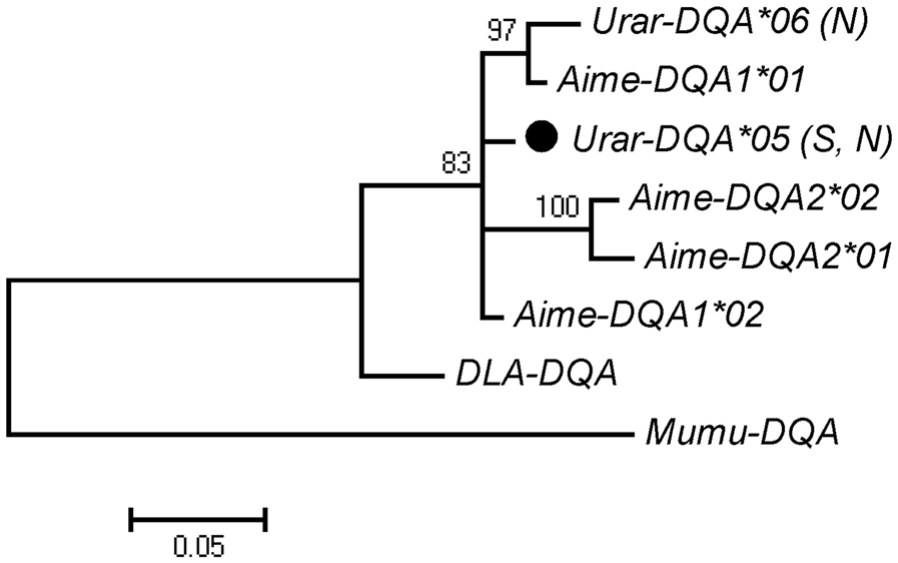
**Phylogenetic tree constructed for a 201 bp fragment of the second exon of MHC class II DQA genes in the Scandinavian brown bear.** Bayesian posterior probabilities above 70% are shown above branches. The allele marked with filled circle is expressed. The other sequences used to generate the tree are: *Ailuropoda melanoleuca*: Aime-DQA1*01 (GQ496171.1), Aime-DQA1*02 (GQ496172.1), Aime-DQA2*01 (GQ496178.1), Aime-DQA2*02 (GQ496179.1); *Canis lupus*: *DLA-DQA* (NM_001011726.1); *Meles meles*: Meme-DQA*01 (HQ908097.1), Meme-DQA*02 (HQ908098.1); *Taxidea taxus*: Tata-DQA*01 (HQ908102). *Mus musculus* sequence: *Mumu-DQA* (V00832.1) was used to root the tree.

Two alleles were found in the 202 bp fragment of DQA 2^nd^ exon in 26 genotyped individuals (Genbank accession numbers JX469890-1). The sequences did not contain indels or PSC. Each allele translated into a unique amino-acid sequence (Figure 1d). All six individuals assayed for expression had only allele Urar-DQA*05 in gDNA and cDNA. Allele Urar-DQA*06 is more similar to one of the giant panda’s alleles than to Urar-DQA*05 (Figure 4). The two nucleotide sequences differed by 2.97%, and the difference at the amino-acid level was 5.97%. d_N_ was not significantly different from d_S_ (Table 4).

## Discussion

We characterised, for the first time, sequences coding for the peptide binding groove (2^nd^ exon) of the MHC class I in the brown bear and report a number of new class II alleles in the Scandinavian brown bear populations. We found abundant variation in sequences coding for peptide binding groove in both MHC classes, but the three analysed class II genes differed in the level of polymorphism. We have found 37 MHC class I, 16 MHC class II DRB (12 of them new), 4 DQB and 2 DQA alleles.

MHC class II genes of the brown bear have been previously studied by Goda *et al*. [35,36]. The authors characterised partial DQA 2^nd^ exon and 2^nd^ intron sequences, reporting 4 alleles containing only nonsynonymous substitutions, predominantly occurring at putative ABS, as inferred from crystallographic models of HLA. These 4 alleles probably represented 2 loci, one of which was not expressed. Our data also suggest the presence of one expressed DQA locus. The rarer of two alleles we found probably belongs to the same locus, as it was not present in six individuals from which we characterised both gDNA and cDNA sequences. Here, we also characterised, for the first time in the brown bear, DQB sequences, encoding the β chain, which forms a biding groove in a dimer with the α chain coded by DQA. The level of polymorphism at DQB was also low, with only four alleles, belonging to two loci, in a sample of 26 individuals. A higher level of polymorphism has been reported for class II DRB 2^nd^ exon, with 19 alleles found in 38 individuals from Japan, Alaska and Siberia [36]. Also Scandinavian populations are characterised by substantial DRB polymorphism, with 16 alleles present, but only 4 alleles were identical to those reported earlier. Goda et al. [36] inferred the presence of at least two DRB loci, but their expression status was not established. Our data confirm this number and confirm that both loci are expressed.

MHC class I in brown bear, characterised for the first time in this study, consist of at least three expressed, and at least two non-expressed loci. Two alleles (Urar-U*01 and *24) clustered with a monomorphic *Aime-152* gene, which suggests that this cluster represents a separate locus in the brown bear. This hypothesis is further supported by presence of at least one sequence belonging to this cluster in all individuals investigated.

The two Scandinavian brown bear populations are highly differentiated in MHC class I genes as well as in class II DRB genes. High F_ST_ in both genes are consistent with findings of Taberlet *et al*. [59], based on mtDNA analysis, that Scandinavian brown bears originated from two refugia and colonized the area from two directions – from the south and the east. Analysis of 19 microsatellites detected three bear subpopulations in Scandinavia: North, Middle and South [60], but confirmed the earlier mtDNA results, in that there was a high genetic differentiation between the south and other two populations. Our samples were from the North and South subpopulations, as defined by Manel *et al.*[60].

MHC class II DRB and DQB genes clustered with respective panda sequences, as expected based on the relative conservation of class II genes among mammals [34]. Non-classical class I brown bear sequences also grouped with the sequence of the non-classical giant panda 1906 locus and with the dog *DLA-79* locus. Another two brown bear sequences formed a distinct cluster with *Aime-152* locus, which was monomorphic in panda. Thus, it seems that orthology has been maintained in MHC class I genes of ursids for over 12 million years, since the divergence of *Ursus* and *Ailuropoda*. Two distinct MHC class I clusters contained non-expressed sequences. The pseudogenisation of a polymorphic cluster probably exemplifies a birth-and-death process, which in the long run may cause the lack of orthology of MHC class I genes among taxa [2,3].

Some examples of trans-species polymorphism were observed between brown bear and giant panda MHC class II sequences DQA (Urar-DQA*06 and Aime-DQA1*01) and possibly also in DRB genes (Urar-DRB*04 and Aime-DRB*02). In class I, alleles Aime-128*03 and Urar-U*29 appeared to group together, but the grouping was only weakly supported. The sparse data does not allow us to establish whether the brown bear MHC class I and class II differ in the extent of transspecies polymorphism, with respect to panda sequences. Transspecies polymorphism was observed for DRB sequences within the genus *Ursus*, as also noted by Goda *et al*. [36]. The lack of sequences for MHC class I did not allow comparison of the extent of transspecies polymorphism at this level between MHC class I and MHC class II.

MHC class II DRB genes showed the strongest signal of historical positive selection. Goda *et al*. [36] also inferred positive selection at ABS sites, but not at non-ABS sites. However, the d_N_/d_S_ ratio they reported (1.96), based on a subset of sequences which we analysed, was substantially lower than our estimate (5.08). In the giant panda, there was an evidence for positive selection at DRB3 locus, with ω estimates of 9.2-10.9, but not at the DRB1 locus [61]. The results for DQB genes showed evidence for positive selection acting only in putative ABS, where d_N_/d_S_ significantly exceeded 1. No signal of positive selection was detected in DQA.

MHC class I genes also evolved under positive selection. Model with positive selection fitted the MHC class I sequences best, and d_N_ at putative ABS sites was twice as high as d_S_, although the excess of non-synonymous substitution was marginally non-significant using the Z test. The d_N_/d_S_ ratio for MHC class I was much smaller than that found for the DRB locus (Table 4). This might be due to including into the analysis pseudogens, which might have lost the signal of positive selection, but excluding pseudogene sequences did not substantially change the estimate. A comparatively lower d_N_/d_S_ ratio in MHC class I could also potentially have resulted from generally higher divergence within MHC class I loci and consequently saturation at nonsynonymous sites [62]. Indeed, d_N_ for MHC class I at ABS was even higher than for DRB genes, but the latter accumulated fewer synonymous substitutions. However, after excluding pseudogenes, d_N_ at ABS for MHC class I was actually lower than for DRB. Thus, selection on DRB loci seems to be more pronounced than that on MHC class I loci, as additionally indicated by a number of positively selected sites (PSS) in DRB exceeding that at MHC class I by a factor of two. As a result, even though the proportion of PSS matching human ABS was actually similar at DRB and MHC class I, as many as 6 PSS were detected outside ABS in DRB, which was also reflected by high d_N_/d_S_ ratios at non-ABS DRB sites.

Very strong positive selection on ABS in DRB was also reported for canines [63], with ω for positively selected sites under M8 model equal to 12.02, a value very similar to the one we found for the brown bear. For comparison, the value is 3.99 for humans and 5.03 in bovines [63]. Thus, it seems that a very strong positive selection on DRB may be a general feature of caniform MHC. The five PSS (positions number 8, 9, 10, 16, 56) which Furlong *et al*. reported as canine-specific (i.e. not overlapping with PSS in primates and bovines) did not overlap with brown bear PSS, except for position 9, which is an ABS. Hence, the strong signal of positive selection detected in brown bear does not seem to result from phylogenetic history, but rather from species-specific selection pressure. Such high selection pressure may be capable of maintaining MHC polymorphism even in heavily bottlenecked populations; indeed an endangered canid, the island fox *Urocyon littoralis* is a rare example of such situation: variation at DRB locus is maintained despite depletion of neutral variation [64]. Simulations have shown, however, that selection pressure from parasites is unlikely to maintain MHC variation in bottlenecked populations [65], so it is tempting to speculate that it may result from mate choice for dissimilar MHC type. We are currently investigating this possibility in the brown bear.

## Conclusion

In summary, our work revealed high polymorphism of both MHC class I and class II DRB genes, with limited polymorphism at DQ genes in two Scandinavian populations of the brown bear. Both MHC class I and DRB genes have undergone significant positive selection during the evolutionary history of brown bear. There were no obvious differences between the classes in the degree of putative orthology to giant panda MHC genes, although pseudogenisation of two of the MHC class I clusters indicated that gene turnover may be higher in this class. Our data provide solid background to study contemporary selection resulting from parasites and mate choice on MHC in the brown bear.

## Competing interests

The authors declare that they have no competing interests.

## Authors’ contributions

KK participated in study design, carried out the laboratory analyses, analysed the data and helped to draft the manuscript, WB helped to analyze the data and to draft the manuscript, KB and EŚ carried some laboratory analyses, JK, PT and JES participated in study design and coordination, provided the samples and commented on the manuscript, JR participated in study design and coordination and helped to draft the manuscript. All authors read and approved the final manuscript.

## Supplementary Material

Additional file 1 **Table S1.** Primer sequences for MHC class I and MHC class II in the brown. Click here for file

Additional file 2 **Supplementary information.** Mammalian MHC sequences used to obtain the brown bear sequences used to design primers. Click here for file

Additional file 3 **Table S2.** The number of MHC class I alleles detected in genomic DNA (gDNA) and transcribed alleles (cDNA). Click here for file

Additional file 4 **Table S3.** The average pairwise nucleotide and amino-acid distances (A), d_N_ and d_S_ for brown bear MHC class I sequences (B). Click here for file

Additional file 5 Nucleotide sequences of MHC class II DRB genes in the Scandinavian brown bear. Click here for file
