# Supplementary material for: Evolution of major histocompatibility complex class I and class II genes in the brown bear
Source: BMC Evol Biol. 2012 Oct 2;12:197. doi: 10.1186/1471-2148-12-197 (PMC3508869; doi:10.1186/1471-2148-12-197)
Supplement: Additional file 1 — Table S1. Primer sequences for MHC class I and MHC class II in the brown. [file 1471-2148-12-197-S1.docx]

**Additional File 1.** Table S1 Primer sequences for MHC class I and MHC class II in the brown bear.

| Primer pair no. | Primer name | Primer sequence (5’-3’) | Gene | Amplicon size without primers (bp) |
| --- | --- | --- | --- | --- |
| 1 | Car_a1F | GGCTCGCACTCCmTGAGGT | MHC class I,  2^nd^ exon | 229 |
|  | Car_a1R | TCGCTCTGGTTGTAGTAGC |  |  |
| 2 | UmarF | GAGTGCCATTTCACCAACGGGA | MHC class II DRB, 2^nd^ exon | 161 |
|  | UmarR | TGTCTGCAGTAGGTGTCCACC |  |  |
| 3 | UmarF | GAGTGCCATTTCACCAACGGGA | MHC class II DRB, 2^nd^ exon | 161 |
|  | UmarRb | TGTCTGCACACTGTGTCCACC |  |  |
| 4 | AidqaL1 | CTGACCATGTTGCTTACTATGG | MHC class II DQA, 2^nd^ exon | 202 |
|  | AidqaR2 | TTGGTAGCAGCGGTATAGTTGGA |  |  |
| 5 | dqbl2 | AGGATTTCGTGyACCAGTTyAAG | MHC class II DQB, 2^nd^ exon | 190 |
|  | dqbr1 | TAGTTGTGTCTGCACACCGTGT |  |  |
