# Supplementary material for: Evolution of major histocompatibility complex class I and class II genes in the brown bear
Source: BMC Evol Biol. 2012 Oct 2;12:197. doi: 10.1186/1471-2148-12-197 (PMC3508869; doi:10.1186/1471-2148-12-197)
Supplement: Additional file 2 — Supplementary information. Mammalian MHC sequences used to obtain the brown bear sequences used to design primers. [file 1471-2148-12-197-S2.docx]

**Additional file 2.** Supplementary information. Mammalian MHC sequences used to obtain the brown bear sequences used to design primers.

Mammalian MHC sequences used to obtain the brown bear sequences used to design primers.

for vectorette PCR (Table S1):

- for MHC class I: *Ailuropoda melanoleuca* (EU162662-81.1), *Canis familiaris* (AF100609.1), *Macaca fascicularis* (HM161393.1),
- for MHC class II DQA: *Ailuropoda melanoleuca* (GQ496171-78.1), *Canis familiaris* (DQ499152.1), *Macaca fascicularis* (HE573233.2), *Zalophus californianus* (AF502564.1),
- for MHC class II DQB: *A. melanoleuca* (GQ496186-8.1), *C. familiaris* (AJ311106.1), *Sus scrofa* (AF464029.1), *Zalophus californianus* (AF503406.1),
- for MHC class II DRB: *A. melanoleuca* (AY895157.1), *C. familiaris* (DQ056280.1), *C. lupus* (DQ056280.1), *Ursus maritimus* (AF458914-38.1),

Mammalian MHC sequences used to design primers used for MHC amplification from cDNA (Table 1):

- for MHC class I: *Homo sapiens* (NM002127.5, NM002116.7, BC007814.2), *Bos taurus* (GQ488024.1, GQ488022.2, NM_001105651.1), *M. nemestrina* (GQ274896.1, GQ274894.1, GQ274890.1, GQ274887.1, GQ274880.1), *A. melanoleuca* (EU162660.1, EU162658.1),
- for MHC class II DQA: *H. sapiens* (NM002122.3, DQ284440.1, DQ284439.1, DQ284438.1), *Aotus nancymaae* (AF201296.1, AF201294.1, AF201293.1), *B. taurus* (NM001012681.1, NM001012675.1, AY730727.1, NM001013601.3), *Equus caballus*: (NM001142814.1, NM001128593.1); *M. fascicularis* (AM943643.2, AM943644.1, AM943642.1), *Ovis aries* (NM_001159759.1, M93433.1, M93431.1); *C. familiaris* (NM001011726.1); *S. scrofa* (DQ159899.1, DQ159895.1, DQ159904.1, NM_001114062.2, DQ159891.1), *Z. californianus* (AF502562.1),
- for MHC class II DQB: *H. sapiens* (NM_002123.4), *B. taurus* (D37952.1, D37954.1, AY730728.1, NM_001034668.2), *M. fascicularis* (AM943645.1), *M. mulatta* (NM_001134297.1), *E. caballus* (L33910.1), *Tursiops truncatus* (EF017815.1), *T. aduncus* (EF507876.1, EF507875.1); *C. familiaris* (NM_001014381.1),
- for MHC class II DRB: *H. sapiens* (NM_022555.3, NM_002124.3, NM_021983.4, M20429.1), *T. truncatus* (EF017817.1, EF507865.1, EF507870.1, EF507867.1), *Canis familiaris* (NM_001014768.1), *B. taurus* (NM_001012680.2), *Rupicapra rupicapra* (AF336340.1), *Capra hircus (*AB008345.1, AB008346.1), *Felis catus* (U51575.1, EU916196.1, EU916197.1, NM_001128072.1), *E. caballus* (NM_001142811.1, NM_001142815.1), *Gorilla gorilla* (M77154.1), *Z. californianus* (AY491459.1), *Hemitragus jemlahicus* (AF336341.1), *Saguinus oedipus* (M76489.1); *S. scrofa* (AB205163.1, NM_001113695.1, AY247785.1), *Callithrix jacchus* (AM114048.1, AM114050.1), *M. fascicularis* (AF492282.1, DQ381750.1, DQ381752.1, DQ381755.2 DQ381756.1), *M. mulatta*: AM690324.1, AM690328.1; *Sciurus aberti* (M97616.1).
