# Supplementary material for: Evolution of major histocompatibility complex class I and class II genes in the brown bear
Source: BMC Evol Biol. 2012 Oct 2;12:197. doi: 10.1186/1471-2148-12-197 (PMC3508869; doi:10.1186/1471-2148-12-197)
Supplement: Additional file 3 — Table S2. The number of MHC class I alleles detected in genomic DNA (gDNA) and transcribed alleles (cDNA). [file 1471-2148-12-197-S3.docx]

**Additional file 3.** Table S2 The number of MHC class I alleles detected in genomic DNA (gDNA) and transcribed alleles (cDNA) (in six bears for which both gDNA and cDNA genotypes were available)**.**

| Idividual | Number of alleles in gDNA | Number of alleles in cDNA |
| --- | --- | --- |
| W212 | 9 | 5 |
| W504 | 7 | 5 |
| W509 | 9 | 5 |
| W624 | 8 | 5 |
| W625 | 8 | 4 |
| W827 | 6 | 3 |
