# Supplementary material for: Evolution of major histocompatibility complex class I and class II genes in the brown bear
Source: BMC Evol Biol. 2012 Oct 2;12:197. doi: 10.1186/1471-2148-12-197 (PMC3508869; doi:10.1186/1471-2148-12-197)
Supplement: Additional file 4 — Table S3. The average pairwise nucleotide and amino-acid distances (A), dN and dS for brown bear MHC class I sequences (B). [file 1471-2148-12-197-S4.docx]

**Additional file 5.** Nucleotide sequences of MHC class II DRB genes in the Scandinavian brown bear.

>*Urar-DRB**11

GTGCGGTACCTGGTCAGAGACATCTATAACGGGCAGGAGAACGTGCGCTTCGACAGCGACGTGGGGGAGTTCCGGGCGGTGACGGAGCTGGGGCGGCCCATCGCTGAGTACCTCAACCAGCAGAAGGACTTCGTGGAGCAGAAGCGGGCCGCGGTGGACACGTACTGCAGACACAACTACGGGGTGTTGGAC

>*Urar-DRB**13

GTGCGGTTCCTGGACAGATACATCTATAACCGGGAGGAGTACGTGCGCTTCGACAGCGACGTGGGGGAGTACCGGGCGGTGACGGAGCTGGGGCGGCACTCCGCTGAGTACTGGAACCAGCAGAAGGACATCATGGAGCGGACGCGGGCCGCGGTGGACACGTACTGCAGACACAACTACGGGGTGTTGGAC

>*Urar-DRB**16

GTGCGGTACCTGCAGAGATACATCTATAACCGGGAGGAGAACGTGCGCTTCGACAGCGACGTGGGGGAGTTCCGGGCGGTGACGGAGCTGGGGCGGCCGGACGCTGAGTACTGGAACCAGCAGAAGGACTTCATGGAGCAGAGGCGGGCCGCGGTGGACAGGTACTGCAGACACAACTACGGGGTGTTCGAC

>*Urar-DRB**17

GTGCGGCTCCTGGTCAGATCCATCTATAACGGGCAGGAGAACGTGCGCTTCGACAGCGACGTGGGGGAGTACCGGGCGGTGACGGAGCTGGGGCGGCCGGACGCTGAGTACTGGAACCAGCAGAAGGACTTCATGGAGCAGAGGCGGGCCGAGGTGGACACGGTGTGCAGACACAACTACGGGGTGTTCGAC

>*Urar-DRB**20

GTGCGGTACCTGGTCAGAGACATCTATAACGGGCAGGAGAACGTGCGCTTCGACAGCGACGTGGGGGAGTTCCGGGCGGTGACGGAGCTGGGGCGGCCCATCGCTGAGTACCTCAACCAGCAGAAGGACTTCGTGGAGCAGAAGCGGGCCGAGGTGGACACGTACTGCAGACACAACTACGGGGTGTTGGAC

>*Urar-DRB**21

GTGCGGCTCCTGGTCAGATACATCTATAACCGGGAGGAGTACGTGCGCTTCGACAGCGACGTGGGGGAGTACCGGGCGGTGACGGAGCTGGGGCGGCCCATCGCTGAGTACTTCAACCAGCAGAAGGACCTCGTGGAGCAGACGCGGGCCGAGGTGGACACGGTGTGCAGACACAACTACGGGGTGTCGGAC

>*Urar-DRB**22

GTGCGGTACCTGGTCAGAGACATCTATAACGGGCAGGAGAACGTGCGCTTCGACAGCGACGTGGGGGAGTACCGGCCGGTGACGGAGCTGGGGCGGCCCATCGCTGAGTACCTCAACCAGCAGAAGGACTTCGTGGAGCAGAAGCGGGCCGAGGTGGACACGTACTGCAGACACAACTACGGGGTGTTGGAC

>*Urar-DRB**23

GTGCGGTACCTGGTCAGAGACATCTATAACCGGGAGGAGTACGTGCGCTTCGACAGCGACGTGGGGGAGTACCGGGCGGTGACGGAGCTGGGGCGGCACTCCGCTGAGTACTGGAACCAGCAGAAGGACATCATGGAGCGGACGCGGGCCGCGGTGGACACGTACTGCAGACACAACTACGGGGTGTTGGAC

>*Urar-DRB**24

GTGCAGTTCCTGGAGAGACACATCTATAACCGGGAGGAGTTCTCGCGCTTCGACAGCGACGTGGGGGAGTACCGGCCGGTGACGGAGCTGGGGCGGCCCATCGCTGAGTACCTCAACCAGCAGAAGGACCTCGTGGAGCAGAAGCGGGCCCAGGTGGACAGGTACTGCAGACACAACTACGGGGTGTCGGAC

>*Urar-DRB**25

GTGCGGTTCCTGGACAGATACATCTATAACCGGGAGGAGTACGTGCGCTTCGACAGCGACGTGGGGGAGTACCGGGCGGTGACGGAGCTGGGGCGGCACTCCGCTGAGTACTGGAACCAGCAGAAGGACATCATGGAGCAGACGCGGGCCGCGGTGGACACGTACTGCAGACACAACTACGGGGTGTCGGAC

>*Urar-DRB**26

GTGCGGCTCCTGGTCAGAGACATCTATAACGGGCAGGAGAACGTGCGCTTCGACAGCGACGTGGGGGAGTTCCGGGCGGTGACGGAGCTGGGGCGGCCGGACGCTGAGTACTGGAACCAGCAGAAGGACCTCGTGGAGCAGAAGCGGGCCGCGGTGGACACGTACTGCAGACACAACTACGGGGTGTCGGAC

>*Urar-DRB**27

GTGCGGTACCTGGAGAGATACATCTATAACGGGCAGGAGAACGTGCGCTTCGACAGCGACGTGGGGGAGTACCGGCCGGTGACGGAGCTGGGGCGGCACTCCGCTGAGTACTGGAACCAGCAGAAGGACATCATGGAGCAGACGCGGGCCGCGGTGGACACGTACTGCAGACACAACTACGGGGTGTCGGAC

>*Urar-DRB**28

GTGCGGTACCTGAACAGATACATCTATAACCGGGAGGAGTACGTGCGCTTCGACAGCGACGTGGGGGAGCACCGGGCGGTGACGGAGCTGGGGCGGCCGGACGCTGAGTACTGGAACCAGCAGAAGGACTTCATGGAGCGGAGGCGGGCCGAGGTGGACACGGTGTGCAGACACAACTACGGGGTGTTCGAC

>*Urar-DRB**29

GTGCGGTACCTGGTCAGAGACATCTATAACGGGCAGGAGAACGTGCGCTTCGACAGCGACGTGGGGGAGTTCCGGGCGGTGACGGAGCTGGGGCGGCCCATAGCTGAGTACCTCAACCAGCAGAAGGACTTCGTGGAGCAGAAGCGGGCCGCGGTGGACACGTACTGCAGACACAACTACGGGGTGTTGGAC

>*Urar-DRB**30

GTGCGGCTCCTGGTCAGAGACATCTATAACGGGCAGGAGAACGTGCGCTTCGACAGCGACGTGGGGGAGTTCCGGGCGGTGACGGAGCTGGGGCGGCCGGACGCTGAGTACTGGAACCAGCAGAAGGACTTCATGGAGCAGACGCGGGCCGCGGTGGACACGTACTGCAGACACAACTACGGGGTGTCGGAC

>*Urar-DRB**31

GTGCGGCTCCTGGTCAGATACATCTATAACCGGGAGGAGTACGTGCGCTTCGACAGCGACGTGGGGGAGTACCGGGCGGTGACGGAGCTGGGGCGGCCCATCGCTGAGTACCTCAACCAGCAGAAGGACCTCGTGGAGCAGACGCGGGCCGAGGTGGACACGGTGTGCAGACACAACTACGGGGTGTCGGAC
