# Supplementary material for: Evolution of major histocompatibility complex class I and class II genes in the brown bear
Source: BMC Evol Biol. 2012 Oct 2;12:197. doi: 10.1186/1471-2148-12-197 (PMC3508869; doi:10.1186/1471-2148-12-197)
Supplement: Additional file 5 — Nucleotide sequences of MHC class II DRB genes in the Scandinavian brown bear. [file 1471-2148-12-197-S5.docx]

**Additonal file 4.** Table S3 The average pairwise nucleotide and amino-acid distances (A), d_N_ and d_S_ for brown bear MHC class I sequences (B). Distances and standard error values are given as percentages per site; standard errors obtained through 1000 bootstrap replicates in parentheses. The distances and standard error values are given as percentages per site; standard errors obtained through 1000 bootstrap replicates in parentheses.

A)

|  | **K2P nucleotide distance** | | | **Poisson corrected amino acid distance** | | |
| --- | --- | --- | --- | --- | --- | --- |
|  | All sites | ABS | Non-ABS | All sites | ABS | Non-ABS |
| Classical loci | 10.2 (1.4) | 31.8 (7.7) | 7.0 (1.2) | 17.4 (3.4) | 60.2 (17.4) | 11.4 (2.7) |
| Non-classical locus | 0.4 (0.4) | 0.0 | 0.5 (0.5) | 1.3 (1.3) | 0.0 | 1.6 (1.6) |
| Pseudogenes | 13.3 (1.8) | 51.5 (17.6) | 8.9 (1.5) | 24.2 (4.3) | 82.8 (27.7) | 17.4 (3.7) |

B)

|  | | **d_N_** | **d_S_** | **Z** | **P** |
| --- | --- | --- | --- | --- | --- |
| Classical loci | All sites | 10.8 (2.2) | 8.7 (2.5) | 0.827 | 0.205 |
|  | ABS | 36.1 (9.8) | 18.5 (12.5) | 1.346 | 0.090 |
|  | non-ABS | 6.9 (1.9) | 7.3 (2.4) | -0.156 | 1.000 |
| Non-classical locus | All sites | 0.6 (0.6) | 0.0 | 1.019 | 0.155 |
|  | ABS | 0.0 | 0.0 | 1.000 | 0.000 |
|  | non-ABS | 0.7 (0.7) | 0.0 | 1.015 | 0.156 |
| Pseudogenes | All sites | 13.2 (2.4) | 13.5 (4.2) | -0.086 | 1.000 |
|  | ABS | 55.8 (23.3) | 31.3 (19.5) | 0.848 | 0.199 |
|  | non-ABS | 8.0 (1.7) | 11.0 (4.0) | -0.817 | 1.000 |
